# Metastatic sites and lesion numbers cooperated to predict efficacy of PD‐1 inhibitor‐based combination therapy for patients with metastatic colorectal cancer

**DOI:** 10.1002/cam4.5959

**Published:** 2023-04-20

**Authors:** Weiqin Jiang, Yinjun He, Wenguang He, Xiang Zhang, Nan Chen, Yandong Li, Weixiang Zhong, Guosheng Wu, Xile Zhou, Hanju Hua, Feng Ye

**Affiliations:** ^1^ Department of Colorectal Surgery, First Affiliated Hospital Zhejiang University School of Medicine Hangzhou China; ^2^ College of Medicine Zhejiang University Hangzhou China; ^3^ Department of Radiology, First Affiliated Hospital Zhejiang University School of Medicine Hangzhou China; ^4^ First Clinical Medical College of Lanzhou University Department of General Surgery Gansu Provincial Hospital Lanzhou China; ^5^ Departments of Colorectal Surgery Yuyao Hospital of Traditional Chinese Medicine Yuyao China; ^6^ Department of Pathology, First Affiliated Hospital Zhejiang University School of Medicine Hangzhou China

**Keywords:** colorectal cancer, combination immunotherapy, liver metastasis, PD‐L1, predictive markers

## Abstract

**Background:**

Limited data have been used to evaluate the efficacy of immunotherapy in metastatic colorectal cancer (mCRC). Furthermore, potential markers that can be used to identify responding patients and to further improve efficacy have not been fully explored.

**Methods and Results:**

In our study, we included a total of 97 patients with mCRC, who each received programmed death‐1 (PD‐1) inhibitor‐based combination therapy at our center. All 12 hypermutated patients benefited from immunotherapy, with median progression‐free survival (mPFS) reaching 28.3 months, regardless of liver metastasis. The objective response rate (ORR) of non‐hypermutated patients was 16.5% (14/85), with an mPFS of 4.0 months. For non‐hypermutated patients, multivariate analysis revealed that the combination of liver metastasis and baseline lesion number significantly stratified response and survival. The lesion‐based analysis indicated that the lymph node was the most responsive, followed by the peritoneum and lung, with liver metastasis being the least responsive. None of the patients (0/7) with negative programmed ligand‐1 (PD‐L1) expression responded, and positive PD‐L1 expression may serve as a biomarker (mPFS 5.7 vs. 2.2 months, *p* = 0.002) that can be used to further guide treatment in non‐hypermutated mCRC with liver metastasis (CRLMs).

**Conclusion:**

Patients with hypermutated mCRC benefited significantly from immunotherapy, whereas the non‐hypermutated cohort with liver metastasis and numerous lesions showed less benefit. The lesion sites reflected varying levels of efficacy, among which PD‐L1 potentially cooperated to guide the immunotherapy of CRLMs.

## 1 INTRODUCTION

Immunotherapy has demonstrated significant clinical benefits in patients with metastatic colorectal cancers (mCRCs) harboring deficient mismatch repair (dMMR) or microsatellite instability high (MSI‐H).^1^, ^2^, ^3^, ^4^ Polymerase ɛ (POLE) mutations, which result in hypermutation in mCRCs in addition to dMMR/MSI‐H status,^5^ are associated with superior efficacy in immunotherapy across all tumors,^6^ although related data regarding mCRCs are limited. Meanwhile, limited antitumor activity has been observed in those with non‐hypermutated tumors though representing 95% of mCRCs. The REGONIVO study^7^ assessing combination therapy with regorafenib and nivolumab, first observed a striking objective response rate (ORR) of 33% in patients with microsatellite stability (MSS) mCRC, although only 24 patients were included. However, a subsequent study evaluating the efficacy of regorafenib and avelumab found no objective response.^8^ Moreover, several exploratory studies investigated the combination of programmed death‐1 (PD‐1) inhibitor with several different tyrosine kinase inhibitors (TKIs) with ORR ranging from 7% to 30%.^9^, ^10^, ^11^, ^12^, ^13^ Thus, the definite efficacy of combination immunotherapy for mCRC has not been established due to small sample sizes.

Furthermore, apart from hypermutated status, potential markers identifying mCRC immunotherapy responders have not been fully estimated. Regarding the discrepancy in response rates in those exploratory studies, liver metastasis is thought to lead to inferior efficacy. Notably, in the REGONIVO study, ORR achieved 50% in patients with lung metastasis compared with only 15% in patients with liver metastasis.^7^ Since then, several studies from China and the United States validated preceding conclusion.^13^, ^14^, ^15^ However, in another retrospective analysis that included 51 Chinese patients treated with combination immunotherapy (fruquintinib, *n* = 28; regorafenib, *n* = 23), only the targeted drug was an independent risk factor for progression‐free survival (PFS) while liver metastasis showed no significant difference.^16^ Additionally, tumor burden, generally represented by the number of metastatic lesions or tumor size, has been reported to predict the efficacy and survival of PD‐1/PD‐L1‐treated patients with various tumor types.^17^, ^18^, ^19^, ^20^, ^21^ However, no studies have assessed its predictive or prognostic value in mCRC receiving immunotherapy. Consequently, existing studies have suggested inferior responses in patients with liver metastasis while characteristics of responders from those with liver metastasis have not yet been identified. Furthermore, other clinical factors including tumor burden and the type of targeted drug, warrant further analysis for their impact on the efficacy of immunotherapy in mCRC.

In this study, we evaluated the efficacy of PD‐1‐inhibitor‐based combination therapy in patients with mCRC and assessed the predictive value of clinical characteristics and biomarkers across multiple dimensions. We sought to identify the markers of responders to further guide clinical practice and improve the efficacy of immunotherapy.

## 2 METHODS

### 2.1 Patients

We conducted a retrospective study to assess the efficacy of combination immunotherapy for mCRC in a real‐world setting. Patients treated with PD‐1‐inhibitor‐based combined therapy (including regorafenib, fruquintinib, and chemotherapy) at the First Affiliated Hospital, Zhejiang University School of Medicine, were included in our study. Only patients who had both baseline measurable lesion(s) and at least one postbaseline radiological disease reassessment were included in the subsequent analysis. Those without baseline radiological assessment or treated with PD‐1 inhibitor monotherapy were excluded from our analysis. To assess the hypermutated status and fully display the clinical characteristics and efficacy of the non‐hypermutated cohort, only patients with identified genomic status (described in the following section) were finally included in our study. Written informed consent was exempt for our study due to the retrospective nature and clinical data were retrieved from patients' medical records supervised by the Research Ethics Committee of the First Affiliated Hospital, Zhejiang University School of Medicine (IIT20210176B). This study was conducted in accordance with the Helsinki Declaration. This trial was registered with Clinicaltrials.gov, NCT05414461.

### 2.2 Response evaluation

The primary outcome of our study was progression‐free survival (PFS). Secondary outcomes included durable clinical benefit (DCB), ORR, disease control rate (DCR), and overall survival (OS). The response was assessed according to immune‐modified response evaluation criteria (imRECIST).^22^ Complete response (CR) was defined as the disappearance of all target lesions and any pathological lymph nodes (whether target or nontarget) must have a reduction in short axis to <10 mm. Partial Response (PR) was defined as at least a 30% decrease in the sum of diameters (SLD) of target lesions, taking as reference the baseline sum diameters. Progressive disease (PD) was defined as >20% increase in SLD compared with the baseline or nadir sum diameters and a subsequent confirmed PD was requested (≥4 weeks). Stable Disease (SD) was defined as neither sufficient shrinkage to qualify for PR nor sufficient increase to qualify for PD, taking as reference the smallest sum diameters while on the study. ORR was defined as the percentage of patients who achieved CR or PR. DCR was defined as the percentage of patients who achieved CR, PR, or SD. DCB was defined as CR or PR or SD that lasted more than 6 months. PFS was defined as the time interval between the initial dose and the first recorded progression or death from any cause. OS was defined as the time from enrollment to death from any cause. The lesion‐based best overall response assessment was consistent with the aforementioned patient‐dependent criteria.

### 2.3 Assessment of tumor burden

Baseline lesion number (BLN) was defined as the total number of both target and nontarget lesions. Maximal tumor size (MTS) was measured as the maximal size of a single target lesion. Summary size of target lesion size (SLS) was determined as the sum of target lesion sizes (the longest diameter for tumor lesions and the size of the short axis of the malignant lymph node).

### 2.4 Assessment of molecular and genomic status

Formalin‐fixed paraffin‐embedded (FFPE) tissue containing histologically confirmed colorectal cancer was retrieved and subjected to immunohistochemistry (IHC) staining with an anti‐human PD‐L1 monoclonal antibody (22C3, Dako) to assess PD‐L1 expression. Considering that the cutoff value of PD‐L1 positivity has not been recognized in mCRC, we referred to a previous study^23^ and defined combined positive score (CPS) ≥10 as PD‐L1 expression positivity. MSI status and genomic variations were detected using ColonCore panel and OncoScreen Plus™ including 41 and 520 genes, respectively. The hypermutated cohort included those harboring either MSI‐H or POLE mutation.

### 2.5 Statistical analysis

Categorical variables were compared in the Chi‐square test or Fisher exact tests. Cutoff values for continuous variables were determined based on the C‐statistic. Continuous variables were compared using the Mann–Whitney test or the Kruskal–Wallis test. PFS and OS were estimated using the Kaplan–Meier method and the difference was tested in a log‐rank test. Multiple factors, including age, sex, primary site, histology, previous therapy, metastatic sites, tumor burden, tumor markers, such as carcinoembryonic antigen (CEA) and carbohydrate antigen (CA19‐9) and combined regimen, were included in the multivariate analysis to assess the association between the baseline clinical characteristic and DCB in non‐hypermutated cohort. Univariate factors with *p* < 0.10 were then examined in a multivariate logistic regression model to further test independence in a forward procedure. A two‐tailed *p*‐value <0.05 was considered statistically significant. Statistical analyses were performed using SPSS statistical software (version 26; IBM) and R (version 3.5.3).

## 3 RESULTS

### 3.1 Patient characteristics

**FIGURE 1:**
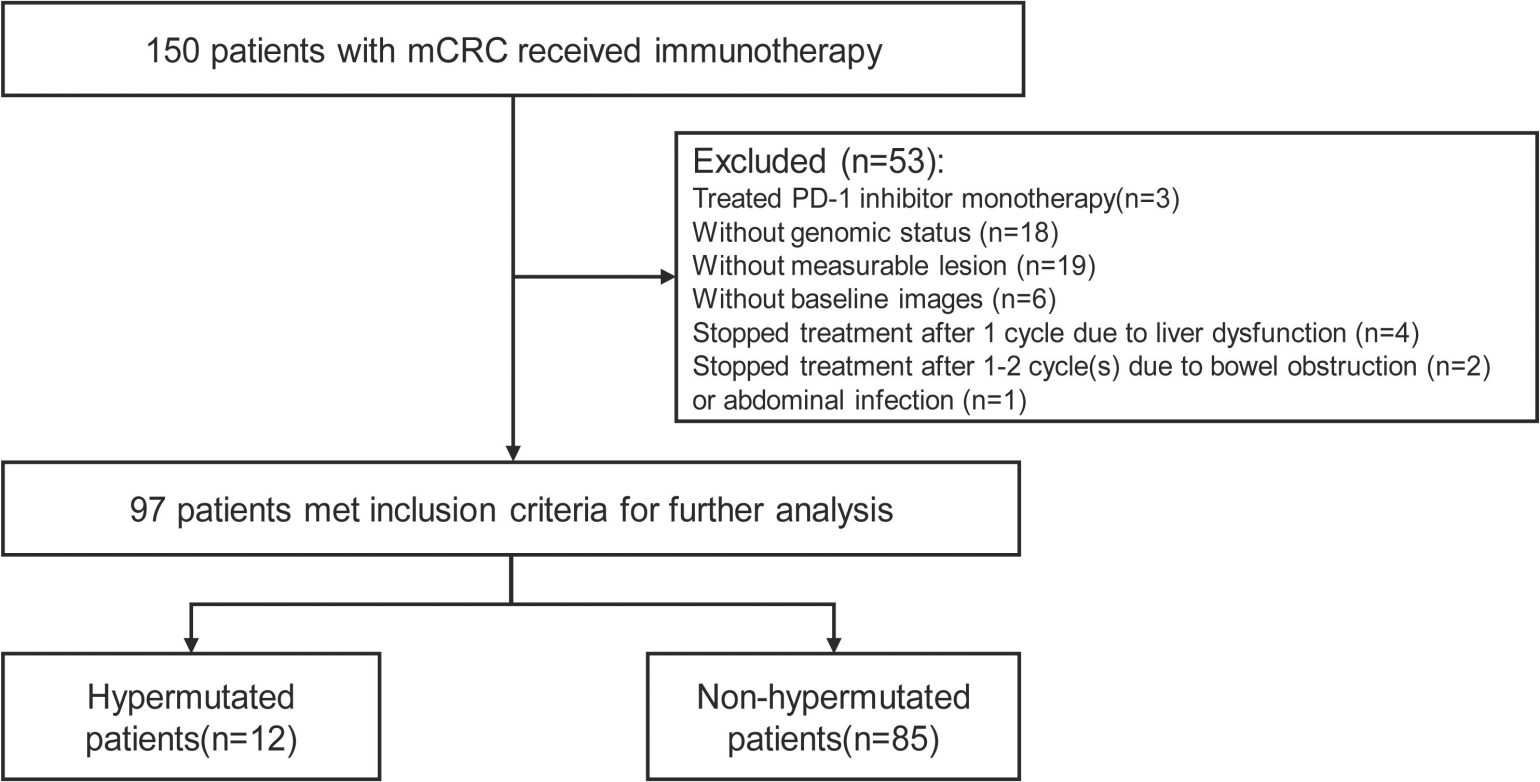
Flowchart of patients with metastatic colorectal cancer (mCRC) receiving immunotherapy.

Between March 10, 2019, and October 22, 2021, we identified 150 metastatic CRC patients who received PD‐1 inhibitor‐based therapy (Figure 1). Three patients with MSI‐H treated with PD‐1 inhibitor monotherapy were excluded from our analysis. Additionally, eighteen patients without identified genomic status, nineteen patients without baseline measurable lesion, and six patients without baseline radiological images were also excluded from this investigation. Seven patients, who discontinued treatment after 1 to 2 cycle(s) due to liver dysfunction (*n* = 4), bowel obstruction (*n* = 2), or abdominal infection (*n* = 1) without radiological reassessment, were also excluded. Finally, we conducted a retrospective analysis on 97 patients including the hypermutated cohort (*n* = 12, 9 MSI‐H and 3 POLE 286R mutations) and the non‐hypermutated cohort (*n* = 85). The median follow‐up was 11.7 months with the final follow‐up date on April 30, 2022. The baseline characteristics of the included patients are listed in Table 1. The included population had a median age of 59 years, while the majority were males (50.5%) and had adenocarcinoma (80.4%). 82 (84.5%) and 12 (12.4%) patients previously received surgery and radiotherapy, respectively. A total of 30 patients (30.9%) had a right‐sided primary tumor in contrast to 66.7% in the hypermutated group. Forty‐nine patients (50.5%) had multiple metastatic sites with liver (54.6%) and lung (50.5%) being the most prevalent. Most patients (80.4%) had more than 5 baseline lesions with a median maximal tumor size of 3.1 cm and a summary target lesion size of 5.6 cm. In the non‐hypermutated cohort, regorafenib was the most commonly used combination regimen (60.0%). In terms of genomic status, thirty‐eight patients had RAS/BRAF V600E wildtype, fifty‐two patients possessed RAS mutations, and seven patients had BRAF V600E mutation.

**TABLE 1:** Baseline clinical characteristics of patients

Characteristics	Total, *n* (%)	Hypermutated group, *n* (%)	Non‐hypermutated group, *n* (%)	*p*‐value
Patients, *N* (%)	97 (100)	12 (12.4)	85 (87.6)	
Median age (range)	59 (26–86)	49 (36–74)	61 (26–86)	0.071
Sex				0.191
Male	49 (50.5)	7 (58.3)	59 (69.4)	
Female	48 (49.5)	5 (41.7)	26 (30.6)	
History of local treatment				
Surgery	82 (84.5)	10 (83.3)	72 (84.7)	1.000
Radiotherapy	12 (12.4)	0 (0)	12 (14.1)	0.356
Location of primary				**0.011**
Right	30 (30.9)	8 (66.7)	22 (25.9)	
Left	67 (69.1)	4 (33.3)	63 (74.1)	
Histology				0.095
AC	78 (80.4)	7 (58.3)	71 (83.5)	
MC or SRCC	19 (19.6)	5 (41.7)	14 (16.5)	
Numbers of metastatic sites				**0.012**
1	48 (49.5)	10 (83.3)	38 (44.7)	
>1	49 (50.5)	2 (16.7)	47 (55.3)	
Metastatic sites				
Liver	53 (54.6)	5 (41.7)	48 (56.5)	0.335
Lung	49 (50.5)	0 (0)	49 (57.6)	**<0.001**
Peritoneum	35 (36.1)	6 (50.0)	29 (34.1)	0.452
Distant lymph node	30 (30.6)	2 (15.4)	28 (32.9)	0.419
Bone	7 (7.2)	1 (8.3)	6 (7.1)	1.000
Baseline lesion number				0.908
≤5	19 (19.6)	3 (25.0)	16 (18.8)	
>5	78 (80.4)	9 (75.0)	69 (81.2)	
MTS (median, cm; range)	3.1 (1.0–14.7)	4.2 (1.5–14.6)	2.94 (1.0–14.7)	**0.024**
SLS (median, cm; range)	5.6 (1.0–25.5)	6.2 (15–25.5)	5.6 (1.0–18.9)	0.222
Numbers of prior treatment lines				**0.005**
≤2	57 (58.8)	12 (100)	45 (52.9)	
>2	40 (41.2)	0 (0)	40 (47.1)	
Combination regimen				**<0.001**
Regorafenib	57 (58.8)	6 (50.0)	51 (60.0)	
Fruquintinib	29 (29.9)	0 (0)	29 (34.1)	
Chemotherapy	11 (11.3)	6 (50.0)	5 (5.9)	
Hypermutated status				**<0.001**
MSI‐H	9 (9.3)	9 (75.0)	0 (0)	
POLE/D	3 (3.1)	3 (25.0)	0 (0)	
MSI‐L/MSS without POLE/D mutation	85 (86.7)	0 (0)	85 (100)	
Genomic status				0.285
RAS/BRAF V600E wildtype	38 (39.2)	5 (41.7)	33 (38.8)	
RAS mutant	52 (53.6)	5 (41.7)	47 (55.3)	
BRAF V600E mutant	7 (7.2)	2 (16.7)	5 (5.9)	

*p*‐value indicated statistically sugnificant *p* < 0.05.

Abbreviations: AC, adenocarcinoma; MC, mucinous adenocarcinoma; MSI, microsatellite instable; MSS, microsatellite stable; MTS, maximal tumor size; SLS, summary size of target lesion size; SRCC, signet‐ring cell carcinoma.

### 3.2 Response and survival

**FIGURE 2:**
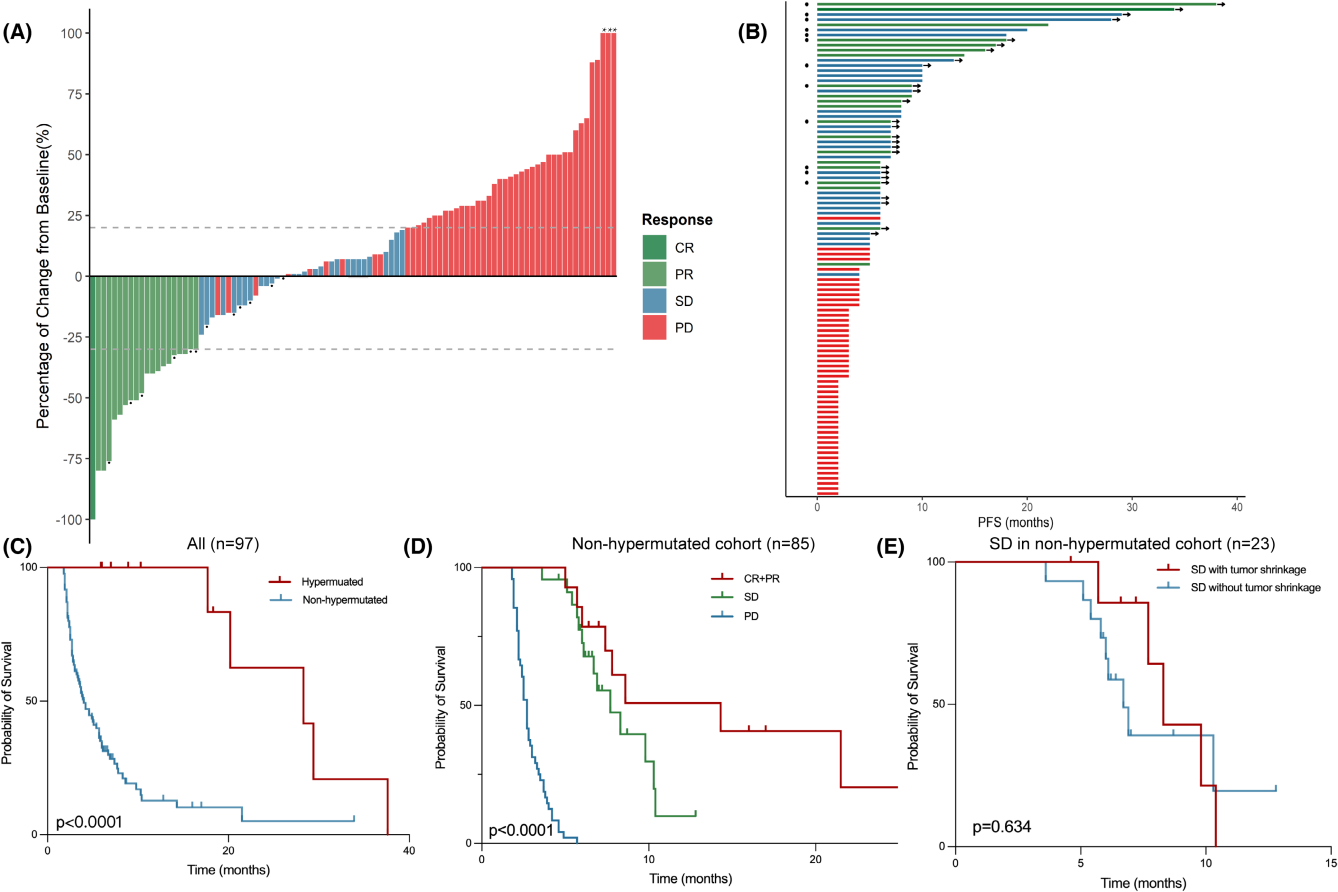
Tumor response assessment with Waterfall plot and Kaplan–Meier plots of progression‐free survival in different groups. Waterfall plot of maximal tumor size change (A) and swimmer plot (B) in hypermutated and non‐hypermutated patients with advanced colorectal cancer. • was annotated for hypermutated patients. * was annotated for three patients with tumor growth exceeding 100%. (C–E) Kaplan–Meier plots of progression‐free survival. (C) Median progression‐free survival was significantly prolonged in hypermutated (*n* = 12) versus non‐hypermutated (*n* = 85) cohorts (28.3 vs. 4.0 months, HR = 0.20, 95% CI 0.12 to 0.34, *p* < 0.0001). (D) Depth of response predicted survival of non‐hypermutated cohort receiving combination immunotherapy. (CR + PR vs. SD vs. PD, 14.3 vs. 7.7 vs. 2.7 months, *p* < 0.0001). (E) No significant difference of survival was observed between subgroups of patients with SD as best response (8.3 vs. 6.7 months, HR = 0.77, 95% CI 0.27 to 2.23, *p* = 0.634). CR, complete response; PD, progressive disease; PR, partial response; SD, stable disease.

In the 97 included patients with measurable lesion(s) and at least one postbaseline radiological disease reassessment, ORR was 20.6% and DCB was 44.3% (Table 2). The median PFS and OS were 5.0 months (95% confidence interval [CI], 3.5–6.5) and 24.1 months (95%CI, 10.5–37.7), respectively. Prolonged mPFS was observed in the MSI‐H cohort compared with the MSS/MSI‐L cohort (28.3 vs. 4.2 months, HR = 6.74, 95% CI 2.72 to 16.69, *p* = 0.001). All 12 patients in the hypermutated cohort achieved durable clinical benefits with median PFS achieving 28.3 months (95% CI, 11.4–45.2). Three patients, including two MSI‐H patients whose best radiological response were PR and SD, respectively, and one patient with POLE mutation who achieved radiological PR, underwent radical resection and were identified to be pathological complete response (pCR). To further investigate clinical benefits except from hypermutated genomic status, we focused on 85 patients in the non‐hypermutated group. Fourteen patients (16.5%) responded to combination immunotherapy with one achieving CR, and the median PFS and OS were 4.0 months (95% CI, 2.9–6.5) and 21.6 months (95% CI, 6.7–36.5), respectively. When the predictive value of depth of response based on RECIST‐based trichotomized response metric was examined, patients with CR/PR (*n* = 14) or SD (*n* = 23) had significantly prolonged survival than those with PD (*n* = 48) (*p* < 0.001). There was no significant difference in mPFS between SD subgroups with or without tumor shrinkage (8.3 vs. 6.7 months, *p* = 0.634) nor between CR/PR and SD groups albeit a trend was observed in the latter two (14.3 vs. 7.7 months, *p* = 0.096) (Figure 2).

**TABLE 2:** Response rate and survival

	Total (*n* = 97)	Hypermutated group (*n* = 12)	Non‐hypermutated group (*n* = 85)
Objective response rate	20 (20.6)	6 (50.0)	14 (16.5)
Complete response	1 (1.0)	0 (0)	1 (1.2)
Partial response	19 (19.6)	6 (50.0)	13 (15.3)
Stable disease	29 (29.9)	6 (50.0)	23 (27.1)
Progressive disease	48 (49.4)	0 (0)	48 (56.5)
Durable clinical benefit	43 (44.3)	12 (100)	31 (36.5)
Median progression‐free survival (months, 95% CI)	5.0 (3.5–6.5)	28.3 (11.4–45.2)	4.0 (2.9–6.5)
Median overall survival (months, 95% CI)	24.1 (10.5–37.7)	29.4 (23.3–35.5)	21.6 (6.7–36.5)

### 3.3 Univariate and multivariate analysis of baseline factors associated with durable clinical benefit in the non‐hypermutated group

**FIGURE 3:**
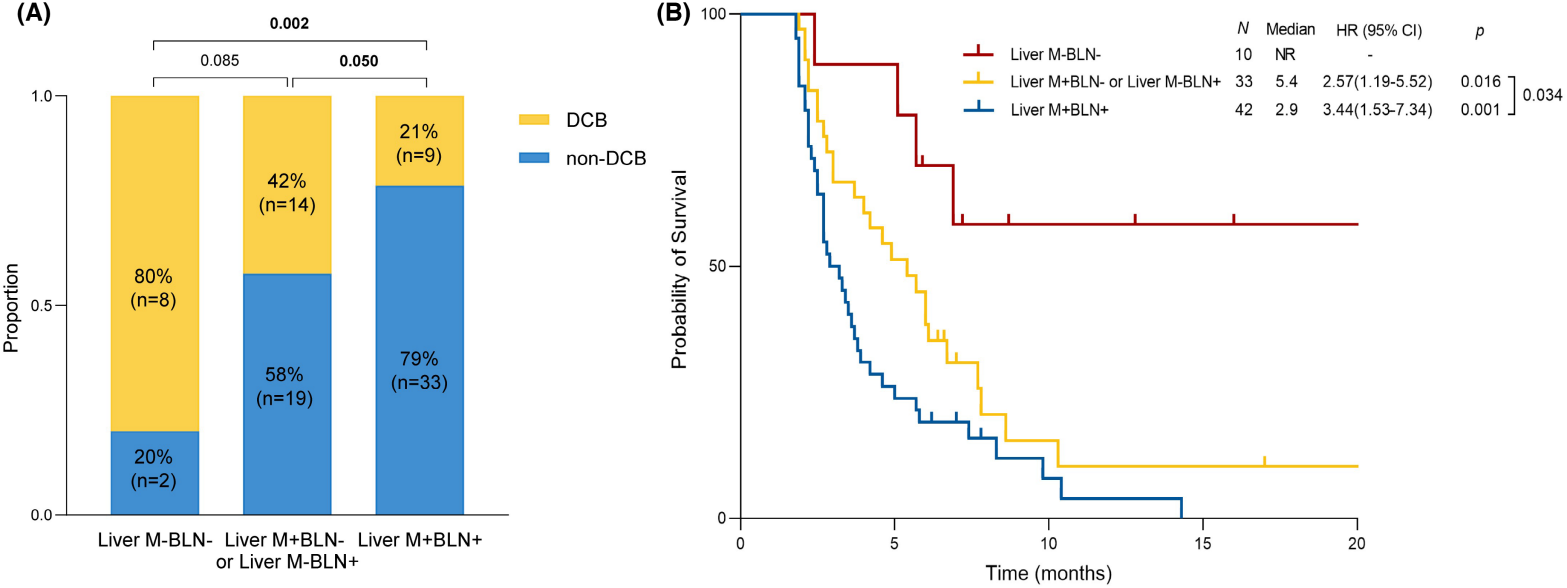
Liver metastasis and baseline lesion number stratified response (A) and progression‐free survival (B) in patients with non‐hypermutated metastatic colorectal cancer receiving combination immunotherapy. BLN, baseline lesion number; DCB, durable clinical benefit; Liver M, liver metastasis; NR, not reached.

Several factors were included in the univariate analysis associated with DCB in the non‐hypermutated group and the results are listed in Table 3. Among them, liver metastasis, more than 2 metastatic sites, more than 5 baseline lesion number, and elevated CEA and CA125 were related to worse DCB and were further estimated in multivariate analysis. Finally, liver metastasis (HR 0.24, 95% CI: 0.09–0.65, *p* = 0.005) and baseline lesion number (HR 3.54, 95% CI: 1.05–11.99, *p* = 0.042) were independently associated with DCB. Additionally, we evaluated the predictive value of combining liver metastasis (Liver M) and baseline lesion number (BLN). We stratified 85 patients with non‐hypermutated mCRC into three subgroups: Liver M^+^BLN^+^ (*n* = 42), Liver M^+^BLN^−^ or Liver M^−^BLN^+^ (*n* = 33), and Liver M^−^BLN^−^ (*n* = 10). The Liver M^+^BLN^+^ group showed the most inferior DCB (21% vs. 42% vs. 80%, *p* = 0.002) and PFS (2.9 months vs 5.4 months vs not reached, *p* = 0.001) than other groups (Figure 3).

**TABLE 3:** Univariate and multivariate association of baseline clinical characteristics with durable clinical benefit (DCB) in non‐hypermutated group

Characteristics	Univariate analysis		Multivariate analysis	
	HR (95% CI)	*p*‐value	HR (95% CI)	*p*‐value
Age (≤60 vs >60)	0.938 (0.387–2.268)	0.886		
Sex (male vs female)	0.554 (0.215–1.426)	0.221		
Primary site (right vs left)	0.994 (0.362–2.725)	0.990		
Histology (AC vs MC + SRCC)	0.511 (0.161–1.625)	0.255		
RAS/BRAF status (wild vs mutated)	0.502 (0.188–1.345)	0.170		
Previous lines of therapy (≤2 vs. >2)	1.085 (0.591–1.995)	0.792		
Liver metastasis (Yes vs No)	0.253 (0.099–0.643)	**0.004**	0.236 (0.085–0.651)	**0.005**
Lung metastasis (Yes vs No)	0.835 (0.342–2.036)	0.691		
Peritoneum metastasis (Yes vs. No)	0.877 (0.343–2.240)	0.784		
Metastatic sites (≤2 vs >2)	2.823 (0.932–8.550)	**0.066**		0.395
Baseline lesion number (≤5 vs. >5)	3.810 (1.225–11.848)	**0.021**	3.544 (1.047–11.989)	**0.042**
Maximal tumor size (≤4 vs. >4 cm)	1.444 (0.518–4.022)	0.483		
Summary size of target lesions (≤7 vs. >7 cm)	2.133 (0.810–5.619)	0.125		
CEA (≤50 vs. >50 ng/mL)	3.274 (1.144–9.370)	**0.027**		0.248
CA19‐9 (≤100 vs. >100 units/mL)	1.991 (0.744–5.332)	0.170		
CA125 (≤35 vs. >35 units/mL)	6.882 (1.464–32.346)	**0.015**		
Combined immunotherapy regimen (Reg vs. Fru vs Chemo)	1.274 (0.581–2.792)	0.545		0.072

*p*‐value indicated statistically sugnificant *p* < 0.05.

Abbreviations: AC, adenocarcinoma; CEA, carcinoembryonic antigen; Chemo, chemotherapy; Fru, fruquintinib; MC, mucinous adenocarcinoma; Reg, regorafenib; SRCC, signet‐ring cell carcinoma.

### 3.4 Lesion‐based response assessment in the non‐hypermutated cohort

**FIGURE 4:**
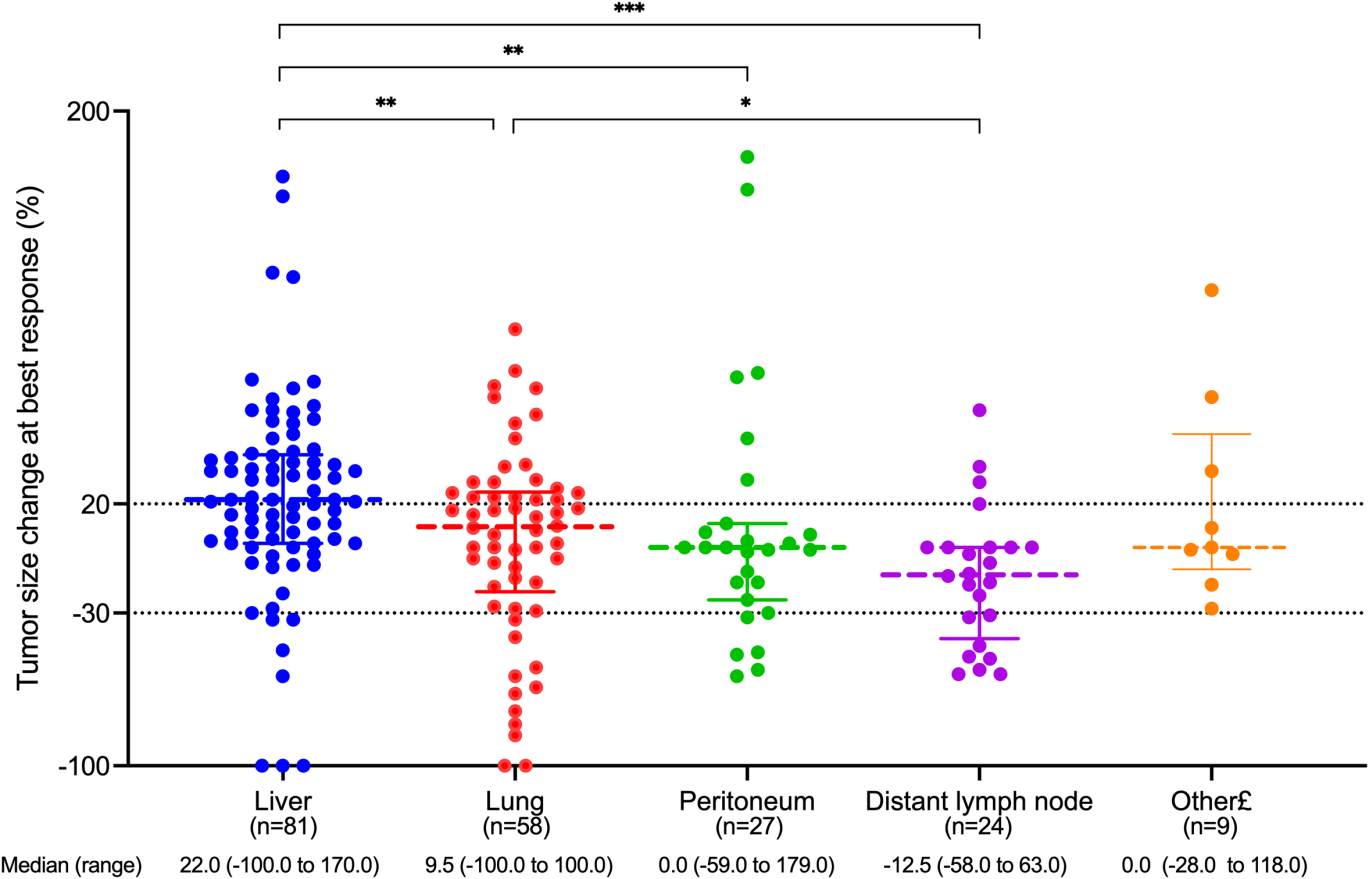
Lesion‐based response to combination immunotherapy in non‐hypermutated cohort. £Other includes adrenal gland (*n* = 2), ovary (*n* = 3), spleen (*n* = 1), muscle (*n* = 1), and local recurrent lesions (*n* = 2) (*p < 0.05, **p < 0.01, ***p < 0.0001).

To further analyze the metastatic site‐based response to combination immunotherapy in the non‐hypermutated cohort, a total of 199 target lesions were included in the lesion‐based response assessment (Figure 4), including 81 liver, 58 lung, 27 peritoneum, 24 lymph node, and 9 other lesions (including 2 adrenal gland, 3 ovary, 1splenic, 1 muscle, and 2 recurrent lesions). Lesion‐based tumor size change at the best response was significantly different across different metastatic lesions (Kruskal–Wallis *p* = 0.001). Liver was the least responsive over other sites compared with lung, lymph node, and peritoneum (*p* = 0.009, *p* = 0.007 and *p* < 0.001, respectively). Lymph node tended to display the most absolute tumor shrinkage over other sites compared with liver and lung (*p* < 0.001 and *p* = 0.025, respectively) while the numeric trend was observed compared with peritoneum though without a statistical difference (the median percentage of tumor size change was −12.5% vs. 0, *p* = 0.155).

### 3.5 Combination of PD‐L1 status stratified efficacy of combination immunotherapy in non‐hypermutated cohort with liver metastasis

**FIGURE 5:**
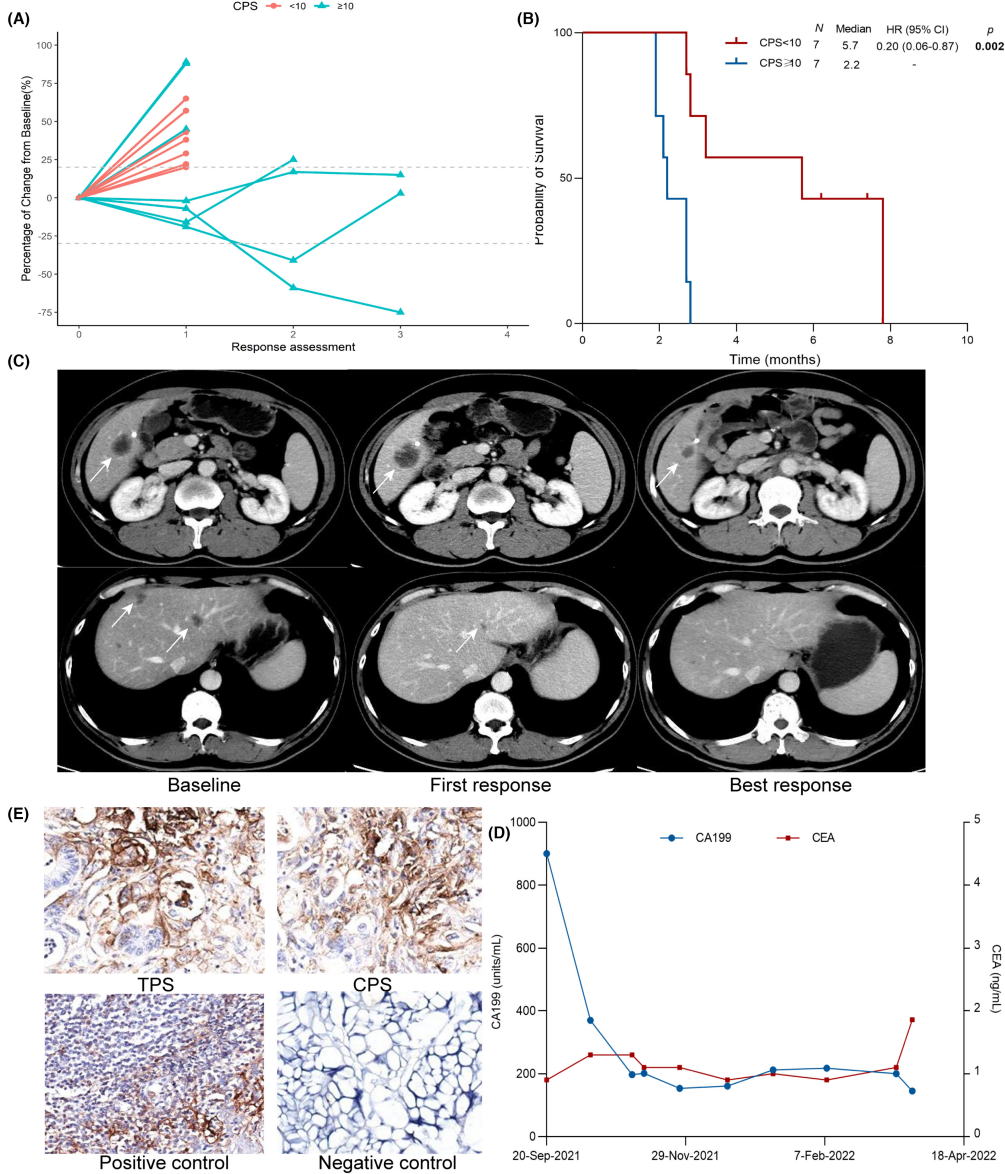
Spider plot of tumor size change over time (A) and Kaplan–Meier plot of progression‐free survival (B) in non‐hypermutated cohort with liver metastasis and different PD‐L1 status (*n* = 14). (C–E) Example of clinical response. The patient with liver metastases (C) and positive PD‐L1 expression (E: TPS 5%, CPS 30) showed an increase in the largest liver lesion while shrinkage in other lesions. (D) CA199 significant declined from 900.7 to 145 units/mL while CEA remained below the upper limit of normal. After first response assessment, the patient experienced durable response in all lesions and was still under treatment up to now.

As aforementioned, the individual‐ and lesion‐based analyses revealed that liver metastasis indicated inferior efficacy of combination immunotherapy in the non‐hypermutated cohort. Non‐hypermutated patients with liver metastasis achieved more inferior DCB (29.7% vs. 54.1%, *p* = 0.004) and PFS (2.9 vs. 6.1 months, *p* < 0.001) than those without liver metastasis. Multivariate analysis suggested no significant correlation between baseline clinical characteristics and the efficacy of combination immunotherapy in non‐hypermutated cohort with liver metastasis (*n* = 48) (Table S1). We further explored other potential biomarkers to guide treatment in this cohort. PD‐L1 status was acquired in 14 patients with liver metastasis and half of them were PD‐L1 positive. While no patient (0/7) with CPS negative and liver metastasis showed response, those with CPS‐positive achieved inspiring response with two PR and one SD (Figure 5). What's more, prolonged median PFS was also observed in the CPS positive cohort (5.7 vs. 2.2 months, *p* = 0.002) though the number of the entire cohort was limited. We displayed an example with liver metastases and positive PD‐L1 expression (TPS 5%, CPS 30) who achieved durable clinical response treated with regorafenib and PD‐1 inhibitor. This 51‐year‐old male had liver, lymph node, and peritoneum metastases whereas target lesions were only observed in liver. After two‐month treatment, this patient experienced an increase in the largest liver lesion while shrinkage in other lesions with a significant CA199 decline and CEA keeping to the normal limit. With SD as the first response assessment, the patient experienced obvious tumor shrinkage of all lesions in the subsequent two radiological assessments. And the patient was still under combination immunotherapy up to now.

## 4 DISCUSSION

In our study, all twelve patients in hypermutated cohort achieved durable clinical benefits though five patients were concomitant with liver metastasis. Hitherto, limited studies reported the efficacy of immunotherapy for those with POLE mutation in mCRC. Michael recently presented a case who had POLE P286R mutation and was treated with pembrolizumab monotherapy obtaining complete remission and a striking four‐year disease‐free survival.^24^ In another study assessing the efficacy of immune treatment in POLE mutations across all tumors, pathogenic POLE mutations, were associated with significant clinical benefits to immunotherapy with DCB reaching 82.4%.^6^ Of three patients with POLE P286R mutation in our study, two received regorafenib and one received capecitabine and bevacizumab as combined regimens when progressed on standard chemotherapy. One patient who achieved PR underwent surgery was demonstrated pCR. Another one achieved durable response after 19‐month regorafenib combined treatment. No radiological tumor shrinkage was observed in the remaining one while persistently stable disease was achieved for more than 1 year. Thus, patients with POLE mutation displayed durable response from immunotherapy. What's more, while the best radiological responses were SD and PR, respectively, in two patients with MSI‐H, pathological complete remission was observed in these two patients and more than two‐year disease‐free interval was achieved. Our results demonstrated absolute benefits of immunotherapy in hypermutated tumors. What's more, as for those hypermutated tumors treated with immunotherapy, radiological assessment even PET‐CT might incorrectly reflect the clinical response, and other biomarkers are warranted to be explored (Figure S1).

Multivariate analysis of our study revealed that the combination of liver metastasis and baseline lesion number significantly stratified response and survival in patients with non‐hypermutated mCRC receiving combination immunotherapy. However, neither RAS mutational status nor targeted drug was demonstrated as an independent risk factor (Table 3). The role of RAS mutation remains controversial in predicting the efficacy of immunotherapy in mCRC. In the Keynote 177 study^1^ and a recently published retrospective study,^14^ RAS mutation was associated with inferior efficacy of immune checkpoint blockades, while no significant difference was observed in another retrospective study.^16^ Moreover, while a preclinical study demonstrated KRAS mutation induced a more suppressive tumor microenvironment,^25^ RAS mutation was associated with an increased proportion of tumor‐infiltrating lymphocytes in the MSS group.^26^ Thus, a large cohort was warranted to further evaluate the role of RAS mutation in mCRC. Besides, with limited studies assessing the efficacy of fruquintinib‐combined therapy, a retrospective study indicated that fruquintinib prolonged the survival of patients with MSS mCRC than regorafenib though only 51 patients were included in this study (fruquintinb cohort, *n* = 28; regorafenib cohort, *n* = 23).^16^ However, no significant difference was observed in our study (fruquintinb cohort, *n* = 29; regorafenib cohort, *n* = 51). More studies are warranted to compare the efficacy of regorafenib and fruquintinib in combination with immunotherapy.

Organ‐based differential tumor response to immunotherapy has been observed across NSCLC,^27^ melanoma^28^ and hepatocellular carcinoma,^29^ among which lung or lymph node lesions were the most responsive. In the REGONIVO study,^7^ patients with lung metastasis showed better response than those with liver metastasis (50% vs. 15%). However, patients with or without lung metastasis showed no difference in response in another fruquintinib‐based study.^30^ While limited data was available to compare responses across different organs in patient‐based analysis, we conducted lesion‐based analysis in the non‐hypermutated cohort to further evaluate various responses across different sites. Lymph node displayed absolute shrinkage over lung and liver (*p* < 0.001 and *p* = 0.025, respectively) while a numerical difference was observed compared with peritoneum lesions. Our study revealed that patients with lymph node metastasis tended to be the most responsive to immunotherapy while liver metastasis in non‐hypermutated tumors indeed indicated resistance to immunotherapy with both inferior DCB (29.7% vs. 54.1%, *p* = 0.004) and PFS (2.9 vs. 6.1 months, *p* < 0.001).

Interestingly, five patients with hypermutated tumor and liver metastasis experienced DCB in our study. According to a lesion‐based study including 78 patients with dMMR solid tumors treated with PD‐1 monotherapy,^27^ most lesions responded irrespective of organ sites and no significant difference was observed. In another study constructing a nomogram to predict the efficacy of immunotherapy in patients with MSI‐H mCRC,^31^ only five variates (regimen, Eastern Cooperative Oncology Group Performance Status, prior lines, neutrophil‐to‐lymphocytes ratio, and palates) from 23 characteristics were demonstrated to independently predict the 12‐month PFS. Unexpectedly, liver metastases showed no significant negative impact on survival, implying that hypermutated genomic status significantly reshaped the tumor microenvironment even in immune privilege organ. Thus, regarding different hypermutated genomic status, disparate mechanisms for treatment resistance and potential biomarkers warrant separate exploration.

With growing evidence suggesting the poor outcomes of immunotherapy in patients with liver metastasis, related studies further evaluated different immune microenvironments in liver metastases from other organs. Discordance of immune contexture between the primary site of colorectal cancer and liver or lung metastatic site has been revealed that PD‐1/PD‐L1 expression was increased in lung metastases compared with liver metastases and primary site.^32^ The expression of PD‐L1 in liver metastases was positively related to the infiltration of CD4‐ and CD8‐positive cells.^33^ However, the correlation between the efficacy of immunotherapy and the expression of PD‐L1 in patients with liver metastases has not been estimated in colorectal cancer. In our study, for the first time, we found that patients with liver metastases and CPS negative did not respond to immunotherapy, while two patients who responded were identified as CPS positive. Significantly prolonged survival was also observed in this cohort (mPFS 5.7 vs 2.2 months, *p* = 0.002). Similar to our results, PD‐L1 expression in ≥25% of tumor cells stratified the survival of patients with NSCLC and liver metastasis receiving immunotherapy.^34^ In another study, positive PD‐L1 expression, the cutoff value of which no matter was 1% or 50%, significantly indicated better DCB of distant metastasis (including bone, brain, adrenal gland, liver and other) in patients with NSCLC.^35^ Together with our study, these results suggested that the non‐hypermutated cohort with liver metastasis and positive PD‐L1 expression were the potential to benefit from immunotherapy though liver metastasis significantly indicated worse efficacy. What's more, those with liver metastasis and negative PD‐L1 expression should be cautious to adopt immunotherapy.

In addition to the disease sites and hypermutated status, exorbitant tumor burden was also found to fail immunotherapy although robust T cell reinvigoration was observed after receiving anti‐PD‐1 therapy.^36^ Tumor burden was a significant factor in clinical decision‐making, with more aggressive treatment being applied to patients with larger tumor burden.^17^ The number of metastatic lesions and tumor size generally represented tumor burden, which was referred from clinical prognostic factors for traditional chemotherapy or targeted therapy, and has been further evaluated as potential predictors in immunotherapy. Baseline lesion number has been found to predict efficacy and survival for advanced gastric cancer in patients receiving immunotherapy while the combination of tumor mutation burden (TMB) further stratified those patients.^21^ Besides, the sum of longest diameters has also been identified as an independent prognostic factor for the survival of PD‐1/PD‐L1‐treated patients in various tumor types.^17^, ^18^, ^19^, ^20^ However, no studies have evaluated the predictive or prognostic value of the aforementioned factors in patients with mCRC receiving immunotherapy so far. Our study suggested that baseline lesion number, rather than tumor size, was an independent prognostic factor for immunotherapy. Combination of baseline lesion number and liver metastasis significantly stratified patients receiving immunotherapy.

However, there are some limitations in our study. First, our sample size was small and there might be some bias in our analysis. Second, due to the retrospective nature of our study, further verification was needed in a large prospective cohort. Third, available patients with concomitant liver metastasis and assessment of PD‐L1 expression were limited and a larger cohort was warranted to provide more detailed immune contexture description for subpopulations of immune cells. Despite these limitations, our study was the largest retrospective immunotherapy study in the Asian mCRC population. We found an absolute benefit of immunotherapy in patients with hypermutated tumor, and the combination of liver metastasis and baseline lesion number significantly stratified the response and survival in non‐hypermutated mCRCs. Furthermore, our data firstly identified patients with liver metastasis who were potentially beneficial from immunotherapy.

## ETHICS APPROVAL AND CONSENT TO PARTICIPATE

This study was supervised by the Research Ethics Committee of the First Affiliated Hospital, Zhejiang University School of Medicine.

## CONSENT FOR PUBLICATION

All authors have made a substantial contribution to this article and are consent for publication.

## AUTHOR CONTRIBUTIONS

**Weiqin Jiang:** Conceptualization (equal); data curation (equal); writing – original draft (equal). **Yinjun He:** Data curation (equal); formal analysis (equal); writing – original draft (equal). **Wenguang He:** Formal analysis (equal); methodology (equal); writing – original draft (equal). **Xiang Zhang:** Data curation (equal). **Nan Chen:** Data curation (equal). **Yandong Li:** Funding acquisition (equal); resources (equal). **Weixiang Zhong:** Methodology (equal). **Guosheng Wu:** Funding acquisition (equal); resources (equal). **Xile Zhou:** Funding acquisition (equal); resources (equal); writing – review and editing (equal). **Hanju Hua:** Funding acquisition (equal); resources (equal); writing – review and editing (equal). **Feng Ye:** Conceptualization (equal); funding acquisition (equal); resources (equal); writing – review and editing (lead).

## FUNDING INFORMATION

The work was supported by the Natural Science Foundation of Zhejiang Province (LGF22H160007, LY22H160045, LD21H030001, LY20H030011), National Natural Science Foundation of China (82170662), and Beijing Xisike Clinical Oncology Research Foundation (Y‐Young2020‐0471).

## CONFLICT OF INTEREST STATEMENT

The authors declare that they have no conflicts of interest.

## Supporting information


Figure S1.

Table S1.

## Data Availability

In order to protect the privacy of the patients, individual data is only available upon reasonable request in accordance to corresponding regulatory.
