# Supplementary figures and images for: Metastatic sites and lesion numbers cooperated to predict efficacy of PD‐1 inhibitor‐based combination therapy for patients with metastatic colorectal cancer
Source: Cancer Med. 2023 Apr 20;12(11):12482–94. doi: 10.1002/cam4.5959 (PMC10278516; doi:10.1002/cam4.5959)

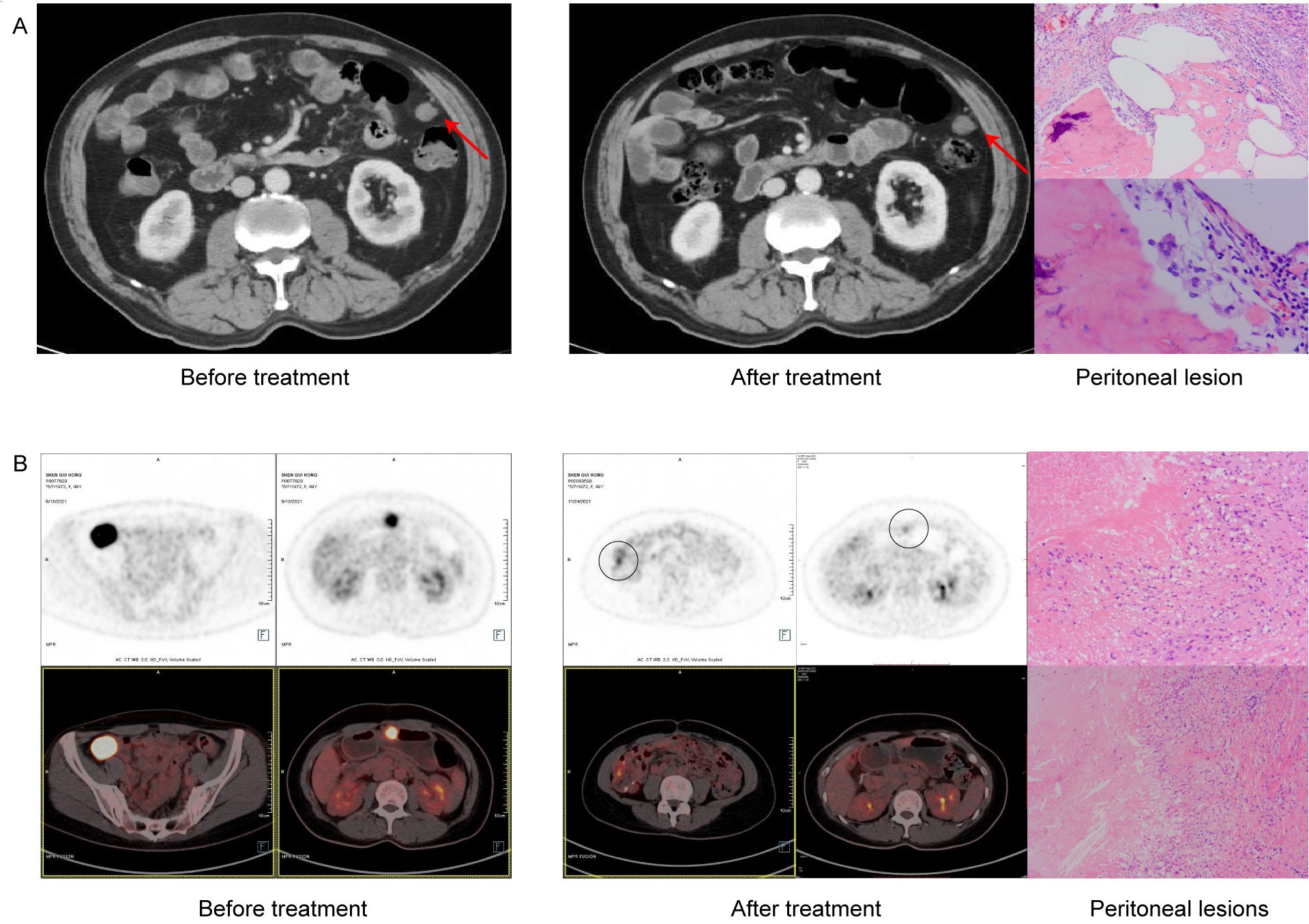

Supplement: Supplementary file 1 — Figure S1. [file CAM4-12-12482-s002.jpg]
