# Supplementary material for: Metastatic sites and lesion numbers cooperated to predict efficacy of PD‐1 inhibitor‐based combination therapy for patients with metastatic colorectal cancer
Source: Cancer Med. 2023 Apr 20;12(11):12482–94. doi: 10.1002/cam4.5959 (PMC10278516; doi:10.1002/cam4.5959)
Supplement: Supplementary file 2 — Table S1. [file CAM4-12-12482-s001.docx]

Supplementary Table 1. Univariate and multivariate association of baseline clinical characteristics with durable clinical benefit (DCB) of patients with liver metastasis in non-hypermutated group (n=48)

| Characteristics | Univariate analysis | |
| --- | --- | --- |
|  | HR (95% CI) | p-value |
| Age (≤60 vs >60)  Sex (male vs female)  Primary site (right vs left)  Histology (AC vs MC+SRCC)  RAS/BRAF status (wild vs mutated)  Previous lines of therapy (≤2 vs >2)  Metastatic sites (≤2 vs >2)  Baseline lesion number (≤5 vs >5)  Maximal tumor size (≤4 vs >4cm)  Summary size of target lesions (≤7 vs >7cm)  CEA (≤50 vs >50 ng/mL)  CA19-9 (≤100 vs >100 units/mL)  CA125 (≤35 vs >35 units/mL)  Combined immunotherapy regimen (Reg vs Fru vs Chemo) | 1.760 (0.453-6.831)  0.331 (0.080-1.372)  0.525 (0.097-2.837)  0.703 (0.116-4.249)  0.643 (0.158-2.609)  1.222 (0.315-4.744)  1.333 (0.332-5.353)  1.833 (0.288-11.665)  2.032 (0.464-8.905)  1.094 (0.283-4.233)  2.353 (0.510-10.859)  1.588 (0.381-6.625)  6.400 (0.723-56.635)  0.835 (0.302-2.311) | 0.414  0.127  0.454  0.701  0.536  0.772  0.685  0.521  0.347  0.897  0.273  0.526  0.095  0.728 |

AC, adenocarcinoma; MC, mucinous adenocarcinoma; SRCC, signet-ring cell carcinoma; CEA, carcinoembryonic antigen; CA, carbohydrate antigen; Reg, regorafenib; Fru, fruquintinib; Chemo, chemotherapy.
